# Antibody Light Chains: Key to Increased Monoclonal Antibody Yields in Expi293 Cells?

**DOI:** 10.3390/antib11020037

**Published:** 2022-05-18

**Authors:** Siqi Gong, Seijal Gautam, Joshua D. Coneglio, Hanna B. Scinto, Ruth M. Ruprecht

**Affiliations:** 1 New Iberia Research Center, University of Louisiana at Lafayette, New Iberia, LA 70560, USA or siqi.gongs@gmail.com (S.G.); seijal.gautam1@louisiana.edu (S.G.); joshua.coneglio1@louisiana.edu (J.D.C.); 2 Department of Microbiology, Immunology & Molecular Genetics, University of Texas Health Science Center at San Antonio, San Antonio, TX 78229, USA; hanna.scinto@nih.gov; 3 Department of Virology and Immunology, Texas Biomedical Research Institute, San Antonio, TX 78227, USA; 4 Department of Biology, University of Louisiana at Lafayette, Lafayette, LA 70503, USA

**Keywords:** monoclonal antibody, recombinant IgM, recombinant dIgA, recombinant IgG, lambda light-chain preference, mAb yield

## Abstract

When constructing isogenic recombinant IgM–IgG pairs, we discovered that μ heavy chains strongly prefer partnering with λ light chains for optimal IgM expression in transiently cotransfected Expi293 cells. When μ chains were paired with κ light chains, IgM yields were low but increased by logs—up to 20,000 X—by using λ chains instead. Switching light chains did not alter epitope specificity. For dimeric IgA2, optimal expression involved pairing with λ chains, whereas light-chain preference varied for other immunoglobulin classes. In summary, recombinant IgM production can be drastically increased by using λ chains, an important finding in the use of IgM for mucosal immunoprophylaxis.

## 1. Introduction

The Food and Drug Administration (FDA) approved the first monoclonal antibody (mAb) for human use, muromonab-CD3 (Orthoclone OKT3), in 1986 [1]; currently, >100 mAb-based therapeutics have been FDA-approved [2], and many more are in the late stages of clinical trials [3]. Antibody-based therapeutics are the fastest growing class of drugs on the market and account for nearly a fifth of new drug approvals each year.

Antibody molecules contain multiple identical heavy and light chains through interchain disulfide bonds. Each of the heavy and light chains is composed of one variable and one constant region. Together, the heavy- and light-chain variable regions, termed antigen-binding fragments (Fabs), are responsible for the specific binding to a molecular target and differ from antibody to antibody. However, the remainder of the amino acid sequences remains constant for antibodies of a given subclass.

Antibodies are divided into distinct classes based on their heavy chains. There are five types of heavy chains—α, γ, δ, ε, and μ—corresponding to five classes of immunoglobulins (Igs): IgA, IgG, IgD, IgE, and IgM, respectively. In humans, IgAs are further divided into two isotypes—IgA1 and IgA2. Likewise, IgG has four isotypes—namely, IgG1, IgG2, IgG3, and IgG4. Igs of different classes contain different numbers of subunits, e.g., IgG contains two pairs of heavy and light chains, while IgM molecules are composed of 10 or 12 pairs given that IgMs exist as pentamers or hexamers.

There are two types of light chains, κ and λ chains, which are shared among all classes of antibodies. In humans, κ light chains predominate, and two-thirds of antibodies contain κ light chains [4].

We have focused on the biological activity of antibodies, especially multimeric Igs in mucosal fluids. Using nonhuman primate (NHP) models, our group was the first to demonstrate that when given mucosally, recombinant monoclonal dimeric IgAs (dIgAs) and IgM protect against mucosal simian–human immunodeficiency virus (SHIV) challenge [5,6]. We directly instilled purified isogenic neutralizing anti-HIV envelope (Env) dIgA1, dIgA2, IgM, and IgG1 mAbs into the rectal cavity 30 min before a single high-dose SHIV intrarectal (i.r.) challenge; control animals only received the i.r. SHIV challenge. IgM and dIgA1 provided high levels of protection.

During the preparation of IgM for the NHP studies, we noticed that IgM yields in the supernatants of transfected cells were significantly higher when λ light chains were involved instead of κ light chains. Therefore, we hypothesized that pairing μ heavy chains with λ light chains improves yields in transiently transfected Expi293 cells. To test this hypothesis, we constructed multiple IgM molecules based on parental monoclonal IgGs. After expressing IgMs with either λ or κ light chains in Expi293 cells, we found that yields of IgM were significantly higher when the λ light chains were used compared to κ light chains regardless of the original light-chain usage of the parental IgG mAbs.

To assess whether this observation was restricted to IgM, we also cloned and expressed isogenic IgG1, dIgA1, and dIgA2 mAbs similarly. We found that dIgA2 also preferred λ light chains when expressed transiently in Expi293 cells. In contrast, the light chain preference for IgG1 and dIgA1 mAbs varied.

This is the first study demonstrating that light-chain usage strongly impacts the yield of recombinant IgM and, to a somewhat lesser degree, that of dIgA2, irrespective of epitope specificity. Our report also provides practical guidance to optimize recombinant mAb production and may assist in the development of antibody therapies.

## 2. Materials and Methods

### 2.1. Construction of Expression Plasmids

IgM, IgG1, dIgA1, and dIgA2 mAb expression plasmids were prepared as follows: First, heavy- and light-chain variable gene fragments of each mAb were synthesized based on the gene sequences from GenBank (For human mAb VRC01 [7], the accession numbers are GU980702 and GU980703; for human mAb PGT121 [8], the accession numbers are JN201894 and JN201911; for human mAb PGT145 [8], the accession numbers are JN201927 and JN201910; for human mAb PGT151 [9], the accession numbers are KJ700290 and KJ700282; for human mAb N49P7 [10], the accession numbers are MG819638 and MG819643; and for human mAb 10E8v4 [11], the accession numbers are KU951247 and KU951251). We also synthesized other heavy- and light-chain variable gene fragments based on published sequences from the literature (human mAb Fm-6 [12] and rhesus monkey mAb 33C6 [13]), and sequencing results of antibody genes of L243 mouse hybridoma cells using primers described by Tiller et al. [14]. Then, these heavy- and light-chain variable gene fragments were cloned in-frame downstream of a leader sequence (MGWSCIILFLVATATGVHS) and upstream of human μ, γ1, α1, or α2 heavy and κ/λ light-chain constant regions (UniProtKB number: human Ig heavy constant μ P01871; human Ig heavy constant γ1 P01857; human Ig heavy constant α1 P01876; human Ig heavy constant α2 P01877; human Ig light constant κ P01834; human Ig light constant λ P0DOY2), respectively. The resulting plasmids carried either heavy- or light-chain genes of each class of mAbs.

Heavy and light chains without variable regions, termed “variableless” constructs, were prepared similarly. The exception was that the leader sequence was cloned directly upstream of and in-frame with human μ, γ1, α1, α2, κ, or λ chain-constant regions, respectively.

### 2.2. Expression of Recombinant mAbs

Antibodies were expressed transiently in Expi293F cells (A14527, ThermoFisher Scientific, Waltham, MA, USA) through cotransfection of heavy (γ1/μ/α1/α2) and light-chain expression plasmids (κ/λ) using ExpiFectamine 293 Transfection Kit (A14525, ThermoFisher Scientific) at a heavy chain (HC):light chain (LC) ratio of 1:1. In the case of IgM and dIgAs, an expression plasmid encoding the human J chain precursor (UniProtKB number:human J chain P01591, Geneva, Switzerland) [5] was also included in the cotransfections at HC:LC:J chain ratios of 5:5:1. Cells were maintained in Expi293 expression medium (A1435102, ThermoFisher Scientific) for four to five days at 37 °C, 8% CO_2_ with continuous shaking at 135 rpm. The culture supernatants were harvested and clarified via centrifugation at 4000× *g* for 30 min and filtered through a 0.22 μm pore size hydrophilic polyethersulfone (PES) membrane.

### 2.3. ELISA

Antibody concentrations were measured by ELISA. Briefly, Nunc MaxiSorp 96-well ELISA plates were coated with 1 µg/mL capture antibody (goat anti-human Fcγ (109-005-170, Jackson ImmunoResearch, West Grove, PA, USA) for IgG; goat anti-human Fcμ (109-005-129, Jackson ImmunoResearch) for IgM; goat anti-human Fcα (109-005-011, Jackson ImmunoResearch) for dIgAs) in 100 µL 0.05 M carbonate–bicarbonate buffer, pH 9.6 overnight at 4 °C, washed 3× with 0.05% Tween 20 in phosphate-buffered saline (0.05% PBS/T), and blocked with 1× casein reagent in PBS (ab171532, Abcam, Cambridge, UK) for 1 h at 37 °C. Then, 100 µL of transfection supernatants diluted serially in 1× casein reagent in PBS were added to duplicate wells and incubated for 1 h at 37 °C. Plates were washed 4x in 0.05% PBS/T, and binding was detected with 100 µL of 0.25 µg/mL of horseradish peroxidase (HRP)-conjugated detection antibody (HRP-goat anti-human Fcγ (109-035-170, Jackson ImmunoResearch) for IgG; HRP-goat anti-human Fcμ (109-035-129, Jackson ImmunoResearch) for IgM; HRP-goat anti-human Fcα (2050-05, SouthernBiotech, Birmingham, AL, USA) for dIgAs). After 1 h of incubation at 37 °C, 3,3′,5,5′-tetramethylbenzidine (TMB) single solution (ThermoFisher Scientific) was added, and the addition of 1 N H_2_SO_4_ terminated the reaction. Plates were read at 450 nm (630 nm as reference) by an 800TS Absorbance Reader (BioTek, Winooski, VT, USA). Antibody concentration was determined with the corresponding standard (VRC01-IgG1 for IgG1; human serum IgM (I8260, Sigma-Aldrich, Saint Louis, MO, USA) for IgM; HGN194-dIgA1 (Humabs BioMed, Bellinzona, Switzerland) for dIgAs).

Antigen-specific mAb concentrations were measured similarly, only instead of capture antibody we coated 100 µL of 1 µg/mL gp120 (SHIV-1157ipd3N4) or SARS spike protein (40150-V08B1, SinoBiological, Beijing, China). Antigen-specific mAb concentration was determined with the corresponding standard using available purified antibodies targeting the same antigen.

In competitive ELISA, a fixed amount of the base antibody supernatant that gave ~1 OD in antigen ELISA by itself was mixed with serially increased amounts of the competitor antibody supernatant. The detection antibody was specific to the base antibody (for IgG1 κ vs. λ, either HRP-goat anti-κ (2060-05, SouthernBiotech) or HRP-goat anti-λ chain (AP506P, Millipore, Burlington, MA, USA) antibodies were used depending on the base antibody; for λ-chain IgM competing with κ-chain IgG1, the HRP-goat anti-human Fcγ was used). The interaction of the base antibody and the target antigen was inhibited by the competitor.

## 3. Results

### 3.1. Recombinant IgMs with λ Light Chains Express Better Than Isogenic IgMs with κ Light Chains in Expi293 Cells

In an earlier study, we showed that mucosally applied recombinant anti-HIV Env IgM prevented SHIV infection after mucosal challenge [6]. This was the first proof-of-concept study to demonstrate the protective role of IgM against mucosal AIDS virus transmission. To prepare the recombinant IgMs [6], we class-switched a panel of IgG mAbs. Parental IgG mAbs of interest targeted HIV Env and included rhesus monkey mAb 33C6 [13], human mAbs VRC01 [7], PGT121 [8], PGT145 [8], PGT151 [9], N49P7 [10], and 10E8v4 [11]. We also class-switched human IgG1 Fm-6, which recognizes the SARS coronavirus 1 spike protein [12], and mouse mAb L243, which is directed against HLA-DR, one of the human major histocompatibility complex (MHC) class II molecules [15]. The heavy and light variable genes of each mAb were cloned in-frame with the human μ and λ/κ chain constant regions, respectively. The light-chain usage of each mAb followed that of the original IgG. We cotransfected the heavy- and light-chain construct pairs with an expression plasmid encoding the human joining (J) chain precursor into Expi293 cells to express each of the recombinant IgM mAbs transiently.

**Figure 1:**
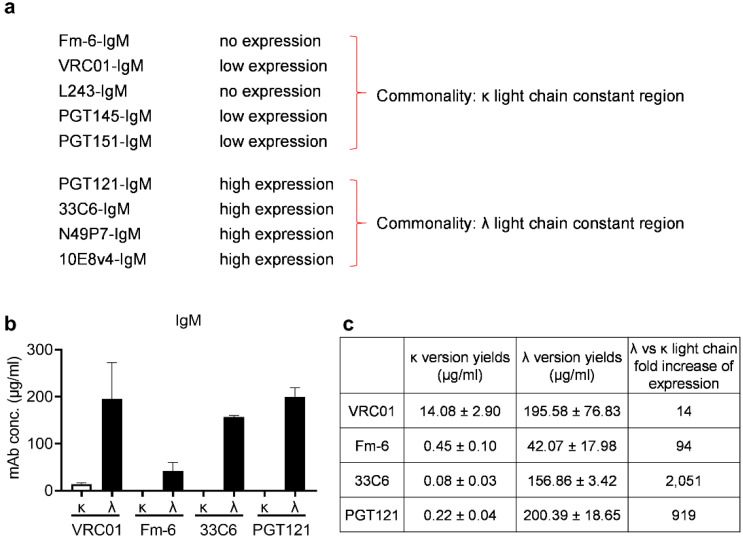
ELISA performed to determine κ versus λ light-chain expression of different recombinant human IgMs obtained after transient transfection in Expi293 cells. Heavy-chain, light-chain (κ or λ), and J-chain constructs were added for the co-transfections: (**a**) the commonality of high expression when λ light chains were paired with µ heavy chains to generate different IgMs; (**b**) the IgM concentrations in culture supernatants of Expi293 cells were determined 5 days post-transfection using ELISA. Error bars represent standard errors of the means. Data are representative of two independent experiments; (**c**) the fold difference when using λ light chains is summarized.

Surprisingly, half of the IgMs gave only minimal yields. We observed that all IgMs with λ light chains expressed highly, while the ones with κ light chains gave poor yields (Figure 1a). Therefore, we hypothesized that μ-chain pairing with λ light chains results in better IgM expression.

To test this hypothesis, we transplanted the light-chain variable genes of VRC01 and Fm-6 from the κ to the λ constant regions. We expressed both versions of each IgM in Expi293 cells and measured the yield of IgMs in culture supernatants. We found that switching from κ to the λ constant regions dramatically increased the yields of recombinant VRC01 and Fm-6 IgMs as well as others (Figure 1b,c, Supplementary Figures S1 and S2).

In addition, we also tested the converse hypothesis: The pairing of μ heavy chains with κ light chains instead of the original λ chains will significantly diminish the yield of recombinant IgMs. To test this notion, we switched 33C6 and PGT121 from λ to κ light chains and expressed both versions of these IgMs in Expi293 cells. As expected, we found that the κ light-chain versions of 33C6-IgM and PGT121-IgM were barely detectable in the transfection supernatants (Figure 1b). The yields were decreased by logs when compared with cotransfections using the λ light chains for these IgM mAbs (Figure 1c). Taken together, our results confirmed that μ-chain pairing with λ light chains gave higher yields in Expi293 cells.

### 3.2. Pairing γ or α Heavy Chains with λ or κ Light Chains Affects the Expression of IgG and Dimeric IgA (dIgA) mAbs in Expi293 Cells

**Figure 2:**
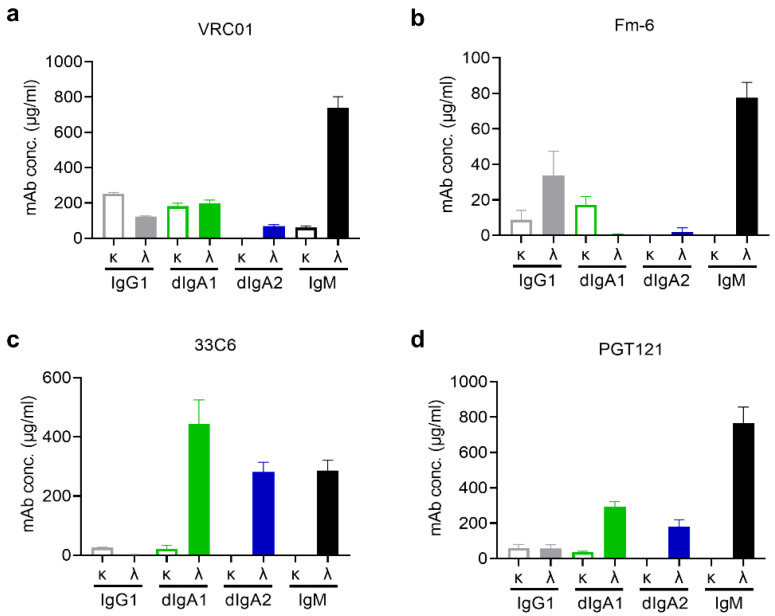
Antigen-specific mAb concentrations of isotypes IgG1, dIgA1, dIgA2, and IgM in culture supernatants of Expi293 cells were determined 5 days post-transfection using ELISA. Four different panels to test the preference for κ versus λ light chains with four different heavy chains were generated for (**a**) VRC01, an anti-HIV Env CD4 binding site mAb [7]; (**b**) Fm-6, an anti-SARS spike protein mAb [12]; (**c**) 33C6, an anti-HIV Env V3 loop mAb [13]; and (**d**) PGT121, a broadly neutralizing anti-HIV Env mAb targeting a complex glycan-dependent epitope [8]. Bars representing IgG1 (grey), dIgA1 (green), dIgA2 (blue), and IgM (black) are either open bars for the κ or solid bars for the λ light-chain versions. Error bars represent standard errors of the means. Data are representative of two independent experiments.

Next, we sought to test whether preference in pairing with λ light chain was restricted to IgM or shared with IgG and dIgAs. To this end, we made human γ1, α1, and α2 heavy chain versions of VRC01, Fm-6, 33C6, and PGT121. We then expressed the recombinant IgG1, dIgA1, dIgA2, and IgM molecules with either λ or κ light chains in Expi293 cells and measured the antigen-specific mAbs in culture supernatants. We found that, for dIgA2, all pairings of the α2 heavy chains with λ light chains produced higher yields compared with pairings with κ light chains (Figure 2, Supplementary Figure S3). The fold differences were dramatic (Table 1), suggesting that λ light chains were preferred for recombinant dIgA2s when expressed in Exp293 cells.

The situation for IgG1 and dIgA1 molecules was not as clear-cut as it was for dIgA2s and IgMs. Pairing with λ light chains was better for producing recombinant Fm-6-IgG1, 33C6-dIgA1, and PGT121-dIgA1, while pairing with κ light chains gave better yields for VRC01-IgG1, Fm-6-dIgA1, and 33C6-IgG1 (Figure 2, Table 1). The remaining VRC01-dIgA1 and PGT121-IgG1 did not show any preference for either λ or κ light chains when expressed in Expi293 cells (Figure 2, Table 1). These data suggested that light-chain usage affected the expression of some but not all recombinant IgG1 and dIgA1 mAbs.

### 3.3. Switching Light-Chain Constant Regions Does Not Change Epitope Specificities

**Figure 3:**
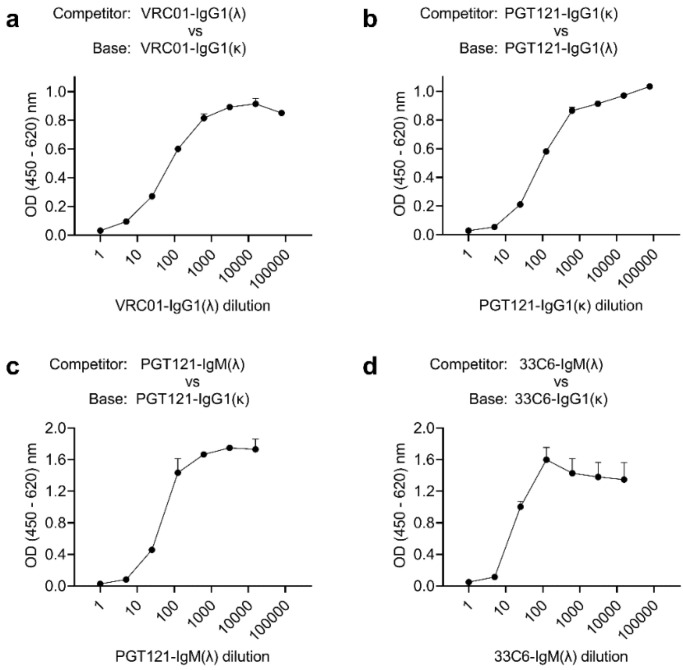
Competition ELISA to assess retention of epitope specificity after switching light chains (Methods): (**a**) testing whether VRC01-IgG1(λ) can compete with VRC01-IgG1(κ); (**b**) testing whether PGT121-IgG1(κ) can compete with PGT121-IgG1(λ); (**c**) testing whether PGT121-IgM(λ) can compete with PGT121-IgG1(κ); (**d**) testing whether 33C6-IgM(λ) can compete with 33C6-IgG1(κ). A dose–response curve is indicative of shared epitope specificity. Data are representative of two independent experiments.

Next, we tested whether switching light-chain constant regions affected the epitope specificities of the resulting mAbs. To this end, we performed competition ELISAs between the κ and λ versions of IgG1 (Figure 3a,b); we found that increasing the competitor concentrations reduced the binding of the corresponding base mAbs. Furthermore, we also used λ versions of IgM as competitors with κ IgG1 versions as base mAbs (Figure 3c,d). The results of this competition ELISA indicate that the same epitopes are recognized by IgG1 and IgM molecules. We concluded that switching light-chain constant regions did not change the binding specificities of the resulting mAbs.

### 3.4. Variable Regions but Not J Chain Also Contribute to Expression Yields

**Figure 4:**
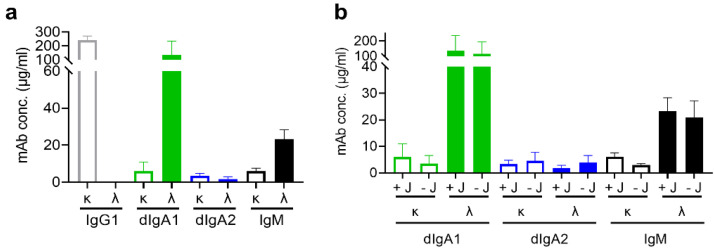
Expression of recombinant mAbs of different Ig classes without variable heavy and light regions. Antibody concentrations in Expi293 cell supernatants were determined 5 days post-transfection using ELISA: (**a**) recombinant mAb levels in culture supernatants when cotransfected with κ or λ light chains lacking variable regions; (**b**) concentrations of recombinant mAbs lacking variable regions in the presence or absence of a J-chain-expressing plasmid. In the absence of J chains, IgAs are expressed in multiple forms [16]. Bars representing IgG1 (grey), IgA1 (green), IgA2 (blue), and IgM (black) are either open bars for the κ or solid bars for the λ light-chain versions. The error bars represent the standard errors of the means. Data are representative of two independent experiments.

Finally, we examined the contributions of variable regions and J chains to the yields of mAbs with κ or λ light chains. To this end, we made constructs to express secreted forms of γ1, α1, α2, μ, λ, and κ chains without variable regions, termed “variableless”. We cotransfected the heavy- and light-chain pairs with the expressing plasmid encoding the human J-chain precursor into Expi293 cells to express such variableless mAbs. As expected, there was a preferred light chain for each variableless Ig class. Pairing γ1 and α2 heavy with κ light chain produced more IgG1 and dIgA2, whereas pairing α1 and μ heavy with λ light chain gave high yields of dIgA1 and IgM (Figure 4a). However, except for IgM, the preference of light-chain usage for variableless antibodies was not consistent with that of the corresponding complete mAbs, suggesting that variable sequences contributed to mAb yield.

We also compared mAb expression levels with or without J chain cotransfection. As shown in Figure 4b and Supplementary Figure S4, the amounts of IgA1, IgA2, or IgM produced with or without the J chain were similar, suggesting that the J chain did not contribute significantly to mAb yields.

## 4. Discussion

In this study, we showed that (1) Ig light chains strongly influenced recombinant monoclonal IgM yields; (2) partnering µ heavy chains with λ light chains increased yields up to 20,000-fold, compared with partnering with κ light chains; (3) switching light-chain usage did not affect epitope specificity; (4) preference in light-chain usage was not restricted to IgM as dIgA2 also preferred λ light chains; (5) for recombinant IgG1 and dIgA1 mAbs, the preference for κ or λ light chains varied.

The genes encoding κ or λ light chains are located on separate chromosomes. When rearrangement happens, κ light chains are generally processed first, then λ light chains [17]. Thus, in most animals, κ light chains are dominant [18,19]. The ratio of κ to λ light chains in murine and human serum are 95:5 and 60:40 [19], respectively. Due to the use of hybridoma technology to generate therapeutic antibodies, κ light chains are also dominant in the FDA-approved mAbs.

However, Mole et al. [4] observed that in human secretions such as saliva, nasal fluids, tears, and fluids produced by glands surrounded by mucosal lymphoid tissues, the κ-to-λ light-chain ratios were lower than those in serum. Therefore, these authors postulated the preferential production of λ light chains in human mucosa. In general, IgAs are the most abundant Ig class in mucosal secretions. IgM, which like dIgAs incorporates the J chain, is also actively transported into mucosal lumina by the polymeric Ig receptor (pIgR). Our finding that λ light chains were preferred for dIgA2 and IgM production coincides with Mole’s finding, suggesting that there may be a benefit of having λ light chains in the mucosal antibodies, such as IgM and dIgAs.

Development and production of therapeutic mAbs are costly, especially for IgM. The high production cost is part of why IgM thus far is not a mainstream therapeutic antibody, even though there is much evidence for IgM’s potential [20]. We have previously shown that 33C6-IgM was more potent than the parental IgG and was highly protective against the mucosal SHIV challenge [6]. Most recently, Ku et al. [21], using anti-SARS-CoV-2 neutralizing mAbs as IgM-14 and IgG-14 versions, also demonstrated the superiority of the IgM isoform: IgM-14 was not only >230-fold more potent in vitro than the parental IgG-14 in neutralizing SARS-CoV-2 but was also active against virus rendered resistant by parental IgG. When administered intranasally to mice as prophylaxis, IgM-14 almost completely blocked lung infection [21].

By using the λ chain constant region to produce IgM in the Expi293 cell-based transient expression system, we showed that the yields of IgM can be increased by logs, compared with the κ-chain versions. This simple change could promote the development of IgM into the frontline and allow the biomedical industry to fully utilize IgM’s advantages in preventing and/or treating illnesses beyond infectious diseases, such as cancer and autoimmune disorders.

In our studies, light-chain preference was not restricted to IgM. We demonstrated that yields of dIgA2 mAbs also increased when λ-chain constant regions were used, while the preference varied among IgG1 and dIgA1 mAbs. However, for most of the mAbs, either λ or κ light-chain constant regions were preferred. Thus, the production of most mAbs can be optimized by testing yields as a function of λ or κ light-chain constant region preference.

This switching of the light chain did not change the binding specificity of an antibody, which is consistent with Montano et al. [22]. These authors demonstrated that the light-chain choice only slightly impacts the structure and functional properties of isogenic IgG. These findings further strengthen the argument to first identify the preferred light-chain constant region for a given mAb before large-scale production instead of just using the original light-chain constant region. As our data indicate, there has been no downside to date when switching the light-chain constant region.

In summary, we demonstrated that switching the light-chain constant region is a simple but excellent way to increase mAb yields, thereby reducing production costs. Our findings could benefit the development of mAbs for prophylactic or therapeutic applications, including combating mucosal pathogens with IgM and dIgA2 mAbs.

## Figures and Tables

**Table 1:** Fold change in expression levels of antigen-specific mAbs when pairing heavy chains with λ versus κ light chains.

	IgG1			dIgA1			dIgA2			IgM		
	Yields (μg/mL)		Fold Difference	Yields (μg/mL)		Fold Difference	Yields (μg/mL)		Fold Difference	Yields (μg/mL)		Fold Difference
	κ	λ	λ/κ	κ	λ	λ/κ	κ	λ	λ/κ	κ	λ	λ/κ
VRC01	252.11 ± 7.09	122.42 ± 3.94	−2	182 ± 17	198.75 ± 17.46	1	2.14 ± 0.3	68.37 ± 8.77	32	60.81 ± 8.29	739.56 ± 62.72	12
Fm-6	8.79 ± 5.38	33.78 ± 13.6	4	17.18 ± 4.73	0.53 ± 0.37	−33	0.09 ± 0.02	1.99 ± 2.28	11	0.004 ± 0.001	77.56 ± 8.67	19,368
33C6	25.1 ± 1.73	1.78 ± 0.4	−14	21.86 ± 11.63	443.96 ± 80.59	20	0.22 ± 0.01	280.7 ± 33.8	1298	0.02 ± 0.01	285.83 ± 35.12	11,630
PGT121	60.45 ± 19.92	56.95 ± 21.38	−1	35.26 ± 5.86	292.72 ± 29.01	8	0.05 ± 0.01	179.37 ± 39.93	3920	0.11 ± 0.01	767.49 ± 89.43	7032

## Data Availability

Not applicable.

## Supplementary Materials

The following supporting information can be downloaded at: https://www.mdpi.com/article/10.3390/antib11020037/s1, Figure S1: The presence of IgM in cotransfection supernatants of Expi293 cells was determined by western blot analysis. (**a**) PGT145-IgM and (**b**) L243-IgM were transiently expressed in Expi293 cells with either κ (left panels) or λ (right panels) light chains. The transfection supernatants were harvested on days 4 and 5 post transfection. The presence of IgM was detected using HRP-goat anti-human Fcμ. The red boxes indicate the migration of polymeric IgM. Figure S2: Representative standard curve for human IgM ELISA. Serially diluted human serum IgM (Sigma-Aldrich) was captured with goat-anti human Fcμ onto an ELISA plate. Then the IgM was detected using HRP-goat-anti human Fcμ. Figure S3. Standard curves for antigen-specific ELISAs. Representative graphs of standard curves for human (**a**) IgG1, (**b**) dIgA1 and dIgA2, and (**c**) IgM. The ELISA plates were coated with SHIV-1157ipd3N4 gp120 or SARS S1 protein. The purified IgG1, dIgA or IgM mAbs recognizing the corresponding target (made in-house) were added at serial dilutions. Then, the mAbs were detected using HRP-goat anti-human Fcγ for IgG; HRP-goat anti-human Fcα for dIgAs and HRP-goat anti-human Fcμ for IgM. Figure S4: Molecular weights of IgMs and IgAs in transfection supernatants of Expi293 cells were determined by western blot. (**a**) IgMs and (**b**) IgAs without variable regions (termed variableless) were cotransfected with or without J chain in the presence of either κ or λ light chain constant regions. The transfection supernatants were harvested on day 5 post transfection. The samples were run under denaturing, non-reducing conditions in 4-12% gradient PAGE gels. The presence of IgM or IgAs was detected using HRP-goat anti-human Fcμ or HRP-goat anti-human Fcα antibody. Red boxes indicate the migration of polymeric and monomeric forms of IgM and IgA.
