# Supplementary material for: Antibody Light Chains: Key to Increased Monoclonal Antibody Yields in Expi293 Cells?
Source: Antibodies (Basel). 2022 May 18;11(2):37. doi: 10.3390/antib11020037 (PMC9149950; doi:10.3390/antib11020037)
Supplement: Supplementary file 1 [file antibodies-11-00037-s001.zip › antibodies-1665581-supplementary.pdf]

Antibody light chains: key to increased monoclonal antibody yields in Expi293 cells?

Siqi Gong[1, 2, 3], Seijal Gautam[1, 4], Joshua D. Coneglio[1], Hanna B. Scinto[2, 3‡], Ruth M. Ruprecht[1, 2, 3, 4*]

1 New Iberia Research Center, University of Louisiana at Lafayette, New Iberia, Louisiana, United States;

2 Department of Microbiology, Immunology & Molecular Genetics, University of Texas Health Science Center at San Antonio, San Antonio, Texas, United States;

3 Department of Virology and Immunology, Texas Biomedical Research Institute, San Antonio, Texas, United States;

4 Department of Biology, University of Louisiana at Lafayette, Lafayette, Louisiana, United States;

‡ Current address: Vaccine Branch, Center for Cancer Research, National Cancer Institute, Bethesda, Maryland, United States;

* Correspondence: ruth.ruprecht@louisiana.edu

Materials and Methods

Western blot

The transfection supernatants were electrophoresed under denaturing, nonreducing conditions in polyacrylamide gels (NuPAGE™ 4-12% Bis-Tris Protein Gel, NP0322, ThermoFisher Scientific or NuPAGE™ 3 to 8%, Tris-Acetate Protein gel, EA0375, ThermoFisher Scientific). Then, the proteins were transferred to nitrocellulose membranes using an iBlot dry blotting system (ThermoFisher Scientific). Finally, the mAbs were detected using horseradish peroxidase (HRP)-conjugated detection antibody [HRP-goat anti-human Fcγ (109-035-170, Jackson ImmunoResearch) for IgG; HRP-goat anti-human Fcμ (109-035-129, Jackson ImmunoResearch) for IgM; HRP-goat anti-human Fcα (2050-05, SouthernBiotech) for dIgAs] in conjuaction with Opti-4CN Substrate Kit (1708235, Bio-Rad) following manufacturer's instructions.

Supplementary Figures

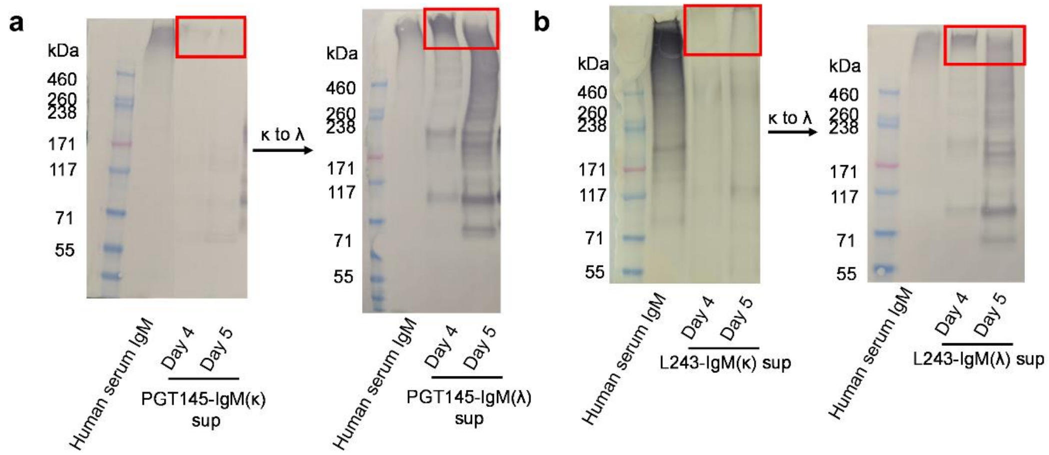

Supplementary Figure S1. The presence of IgM in cotransfection supernatants of Expi293 cells was determined by western blot analysis. (a) PGT145-IgM and (b) L243-IgM were transiently expressed in Expi293 cells with either κ (left panels) or λ (right panels) light chains. The transfection supernatants were harvested on days 4 and 5 post transfection. The presence of IgM was detected using HRP-goat anti-human Fcμ. The red boxes indicate the migration of polymeric IgM.

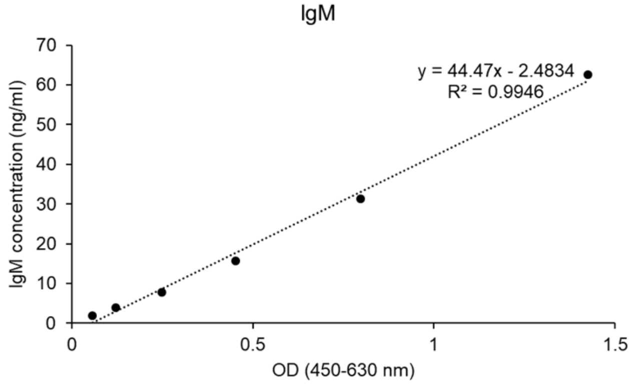

Supplementary Figure S2. Representative standard curve for human IgM ELISA. Serially diluted human serum IgM (Sigma-Aldrich) was captured with goat-anti human Fcμ onto an ELISA plate. Then the IgM was detected using HRP-goat-anti human Fcμ.

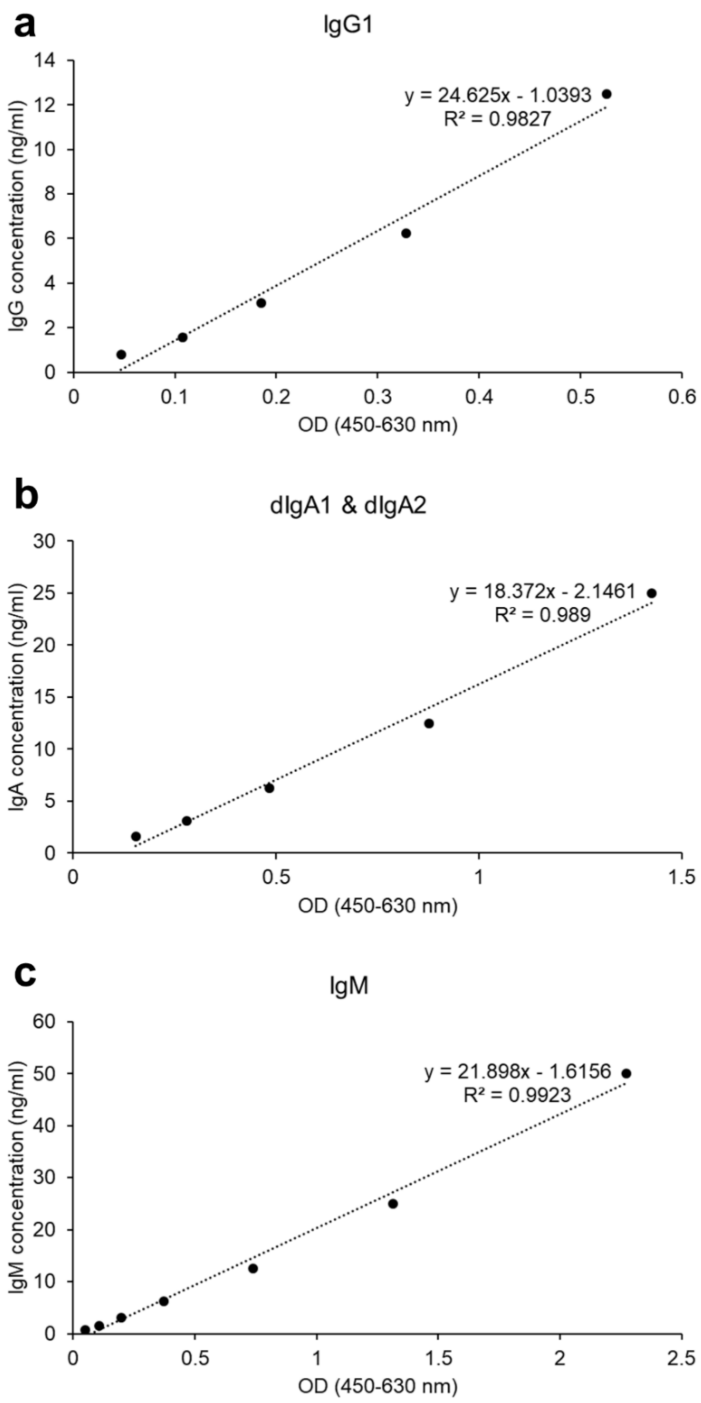

Supplementary Figure S3. Standard curves for antigen-specific ELISAs. Representative graphs of standard curves for human (a) IgG1, (b) dIgA1 and dIgA2, and (c) IgM. The ELISA plates were coated with SHIV-1157ipd3N4 gp120 or SARS S1 protein. The purified IgG1, dIgA or IgM mAbs recognizing the corresponding target (made in-house) were added at serial dilutions. Then, the mAbs were detected using HRP-goat anti-human Fcγ for IgG; HRP-goat anti-human Fcα for dIgAs and HRP-goat anti-human Fcμ for IgM.

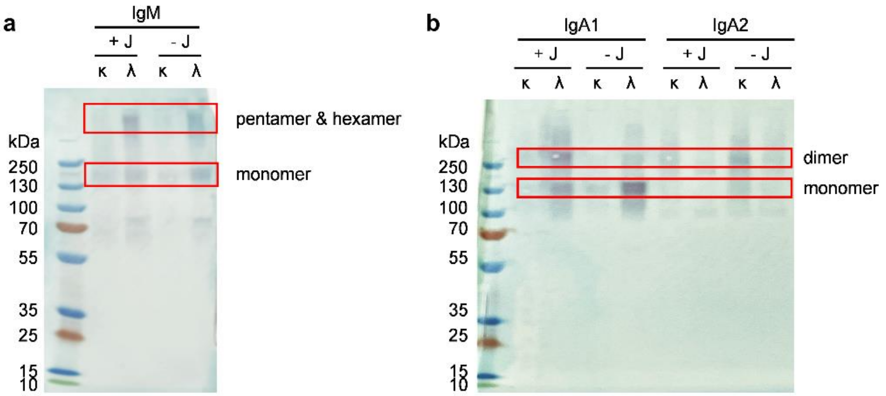

Supplementary Figure S4. Molecular weights of IgMs and IgAs in transfection supernatants of Expi293 cells were determined by western blot. (a) IgMs and (b) IgAs without variable regions (termed variableless) were cotransfected with or without J chain in the presence of either κ or λ light chain constant regions. The transfection supernatants were harvested on day 5 post transfection. The samples were run under denaturing, non-reducing conditions in 4-12% gradient PAGE gels. The presence of IgM or IgAs was detected using HRP-goat anti-human Fcμ or HRP-goat anti-human Fcα antibody. Red boxes indicate the migration of polymeric and monomeric forms of IgM and IgA.
